# Assessing the Knowledge of Diabetic Patients About Glucagon Use in Hypoglycemia: A Cross-Sectional Study in Riyadh, Saudi Arabia

**DOI:** 10.7759/cureus.85017

**Published:** 2025-05-29

**Authors:** Adel Hussien, Alhasan M Alkharasani, Zaid A Alaboudi, Saad A Alkhathran, Talal Abu Suliman, Abdulmohsen F Alanazi, Maymunah I Ezzi, Ghadir J Almajid, Zainab H Almutleeg, Munira A Msawa

**Affiliations:** 1 Medical Biochemistry, Dar Al Uloom University, Riyadh, SAU; 2 College of Medicine, Dar Al Uloom University, Riyadh, SAU

**Keywords:** diabetes, glucagon, hypoglycemia, knowledge, type 1 diabetes, type 2 diabetes

## Abstract

Background and aim

Glucagon is a critical intervention for severe hypoglycemia in diabetic patients. However, knowledge about its use remains insufficient among patients. This study aimed to assess the knowledge level related to glucagon in diabetic patients in Riyadh, Saudi Arabia.

Methods

This cross-sectional survey was conducted among diabetic patients aged 18 years and above in Riyadh, Saudi Arabia. Participants were recruited via social media, diabetes mellitus support groups, and healthcare facilities. The questionnaire collected demographic data, diabetes-related information, and knowledge regarding glucagon use.

Results

The study included 643 respondents, with a majority from the 24 to 34-year age group. Males constituted 60.3% (n=388) of the participants' population. The majority of participants (70.6%, n=454) had type 2 diabetes mellitus (T2DM), whereas 29.4% (n=189) had type 1 diabetes mellitus (T1DM). The majority of respondents stated that they had experienced hypoglycemia (58.3%, n=375), with 43.2% (n=278) experiencing severe hypoglycemia. A total of 69.2% (n=445) of participants were not aware of glucagon injection. Out of those who were aware, 34.3% (n=68) did not have glucagon injection at home, and 17.7% (n=35) were unaware of how to use it. Knowledge of glucagon was higher among younger individuals, those with higher education, and employed participants (p<0.05). T1DM patients showed significantly greater knowledge of glucagon compared to those with T2DM (p<0.05). Those with diabetes for less than five years were more likely to know about the use of glucagon (p<0.05).

Conclusions

There is a significant gap in glucagon knowledge and availability among diabetic patients in Riyadh. Therefore, diabetic patients should be educated about glucagon, especially those with a longer duration of diabetes.

## Introduction

Diabetes mellitus (DM) remains a significant concern globally. Over the years, the prevalence of diabetes has risen considerably, particularly in Saudi Arabia, with approximately 16.4% of the population suffering from diabetes [1]. The management of diabetes poses several challenges due to the short-term and long-term complications associated with it. Among various challenges, hypoglycemia stands out as a critical concern. Hypoglycemia is characterized by low blood glucose concentration, which, if not managed promptly, can lead to cognitive impairment, unconsciousness, brain damage, and even death [2]. Hypoglycemia is more commonly observed in patients with type 1 diabetes (T1DM) compared to type 2 diabetes (T2DM), as individuals with T1DM are typically dependent on insulin or insulin secretagogues, which increase the risk of low blood glucose levels [3]. Globally, the prevalence of hypoglycemia in diabetic patients varies from 14.5 to 42,890 episodes per 1,000 person-years in T1DM and 0.074 to 16,360 episodes per 1,000 person-years in T2DM [4]. In Saudi Arabia, the prevalence of hypoglycemia is 82.5% in T1DM and 12.5% in T2DM [5]. Hypoglycemia is categorized into three levels based on the severity of blood glucose concentration. Level 1 hypoglycemia is characterized by blood glucose concentration less than 70 mg/dL but higher than 54 mg/dL, with patients experiencing dizziness, anxiety, hunger, and palpitation [6]. Blood glucose concentration less than 54 mg/dL is categorized as level 2 hypoglycemia, with clinical manifestations including blurred vision, convulsions, speech disturbance, tremor, and unconsciousness [7]. Level 3 hypoglycemia is a severe form of hypoglycemia and results in cognitive impairment and physical disability. Therefore, such patients require immediate management to improve blood glucose levels [8].

To treat hypoglycemia, patients who are conscious and able to swallow are typically given 15-20 g of quick-digesting carbohydrates. This is particularly done in level 1 hypoglycemia. However, if the patient is conscious but confused, with symptoms like blurred vision, the first step is to administer 15 g of glucose via a glucose tube. If the patient’s condition does not improve and the patient becomes unconscious, then glucagon is injected intramuscularly (IM) [7]. Glucagon is a peptide hormone produced by alpha cells of the pancreas, which regulates blood glucose level by increasing the production of glucose in the liver, neutralizing insulin’s effect [9]. In clinical settings, intravenous (IV), subcutaneous (SC), and IM glucagon are available for the treatment of mild-to-severe hypoglycemia. In non-clinical settings, during severe hypoglycemia when the patient cannot consume glucose orally, the preferred route of injection of glucagon is SC or IM [10]. Many clinical studies have demonstrated the efficacy of glucagon in raising blood glucose concentration in healthy and hypoglycemic diabetic patients in hospital and pre-hospital settings [11]. The intranasal (IN) route is also considered safe for administration during emergency situations where IV access may not be rapidly available [12].

American Diabetes Association (ADA) recommends the utilization of glucagon in all individuals who are at clinical risk of hypoglycemia (<54 mg/dL) [13]. Despite this recommendation, the rate of glucagon utilization and prescription is low. One study investigated the knowledge, possession, and utilization rate of glucagon in diabetic patients and found that out of 300 participants, 55.3% had knowledge about glucagon, 44.7% purchased it, and 35.6% utilized glucagon during hypoglycemic conditions [14]. Conventional glucagon kits are available for ambulatory treatment of hypoglycemic patients, but these kits are complicated and have a lengthy injection method, which can be hard to perform. This may be another contributing factor to low glucagon utilization [15]. Currently, previous studies have reported that glucagon knowledge in the general public is often limited. In Saudi Arabia, there is a paucity of research in this regard that has particularly focused on glucagon knowledge in the diabetic population. This study aimed to assess the knowledge of glucagon use by diabetic patients in hypoglycemic conditions across Riyadh in Saudi Arabia. The findings of this study will pave the way for future guidelines.

## Materials and methods

Study design

This research employed a cross-sectional survey design to assess the knowledge, ability to administer, and incidence of administering glucagon during hypoglycemic episodes among diabetic patients in Riyadh, Saudi Arabia.

Study setting and participants

The study targeted DM patients residing in Riyadh, the capital city of Saudi Arabia, with a population of approximately 8.2 million [16]. A non-probability convenience sampling approach was employed to recruit participants. An online/mobile-based approach was used, and participants were recruited through social media platforms, diabetes support groups, and healthcare facilities. Inclusion criteria encompassed individuals aged 18 years and above, diagnosed with either T1DM or T2DM, and residing in Riyadh. Exclusion criteria included individuals with cognitive impairments or language barriers that hindered survey completion.​ Cochran's formula was utilized to calculate the sample size for a proportionate population, with a 95% confidence level (Z=1.96), a margin of error of 5% (E=0.05), and an estimated proportion (p) of 0.5 to maximize sample size. Considering a 20% non-response rate, the adjusted sample size was approximately 460 participants.​

Data collection tool

The primary data were gathered using a structured questionnaire originally developed for the study by Vilovic et al. [14]. We obtained permission from the original authors to use the questionnaire for our study [14]. The questionnaire consisted of 21 items, divided into three main sections (table in appendix). Demographic details included age, gender, marital status, education level, employment status, income, and residence province. Diabetes-related information included the type of diabetes, duration of diabetes, insulin usage, and experience with low and severe blood glucose episodes. Glucagon knowledge-related information included awareness of glucagon, possession of glucagon, knowledge of glucagon usage, and whether a doctor had recommended glucagon. The questionnaire was pre-tested among a small group of diabetic patients to ensure clarity and reliability. Feedback was used to refine the instrument before the main data collection.​

Data collection procedure

The online questionnaire was distributed through various channels, including social media platforms, diabetes support groups, and healthcare facilities in Riyadh. Participants were informed about the study objectives, and informed consent was obtained electronically before survey participation. To prevent duplicate responses, each Internet Protocol (IP) address was restricted to one submission. Data collection occurred over a period of two months.​

Data analysis

Descriptive statistics were used to summarize demographic and diabetes-related characteristics. Categorical variables were presented as frequencies (n) and corresponding percentages (%). Chi-square tests were conducted to assess associations between glucagon knowledge and various demographic and diabetes-related variables. A p-value of less than 0.05 was considered statistically significant. Data were analyzed using SPSS version 28.0 (Armonk, NY: IBM Corp.) and further managed using Microsoft Excel 2010 (Redmond, WA: Microsoft Corp.).

Ethical considerations

This study adhered to ethical standards, ensuring participant confidentiality and voluntary participation. Ethical approval was obtained from an institutional review board.

## Results

Table 1 shows demographic distribution of the respondents. In terms of age, the majority of respondents are between 24 and 34 years (n=224, 34.8%), followed by those aged 18-24 years (n=98, 15.2%). A smaller percentage is found to be under 18 years (n=13, 2.0%) and 65 years and above (n=33, 5.1%) categories. The sample was predominantly male (n=388, 60.3%), with females making up 39.7% (n=255). Regarding marital status, most respondents are single (n=292, 45.4%), followed by married individuals (n=258, 40.1%), while a smaller proportion are divorced (n=29, 4.5%) or widowed (n=64, 10.0%). In terms of education, the majority hold a bachelor’s degree (n=334, 51.9%), followed by those with a diploma (n=103, 16.0%) or high school education or less (n=95, 14.8%). A smaller percentage have a master’s degree (n=77, 12.0%) or a doctorate or higher (n=34, 5.3%).

Employment status shows that most respondents are employed either full‑time (n=217, 33.7%) or part‑time (n=195, 30.3%), with fewer identifying as students (n=39, 6.1%), retired (n=56, 8.7%), or unemployed (n=94, 14.6%). When it comes to income, most respondents earn between 10,000 and 15,000 (n=320, 49.8%), followed by those earning between 5,000 and 9,999 (n=160, 24.9%). Fewer individuals earn less than 5,000 (n=65, 10.1%) or more than 15,000 (n=98, 15.2%). As for location, the majority reside in suburban areas (n=321, 49.9%), with a significant portion living in urban areas (n=239, 37.2%) and fewer in rural areas (n=83, 12.9%). In terms of household composition, most people live with one to three individuals (n=307, 47.7%), followed by those living with four to six individuals (n=168, 26.1%), and a smaller number living alone (n=112, 17.4%) or with more than six individuals (n=56, 8.7%).

**Table 1:** Demographic details of the participants.

Variable	Category	n	%
Age	Under 18 years	13	2.0
	18-24 years	98	15.2
	24-34 years	224	34.8
	35-44 years	146	22.7
	45-54 years	68	10.6
	55-64 years	61	9.5
	65 years and above	33	5.1
Gender	Male	388	60.3
	Female	255	39.7
Marital status	Single	292	45.4
	Married	258	40.1
	Divorced	29	4.5
	Widowed	64	10.0
Education	High school or less	95	14.8
	Diploma	103	16.0
	Bachelor degree	334	51.9
	Master’s degree	77	12.0
	Doctorate or higher	34	5.3
Employment	Employed full time	217	33.7
	Employed part time	195	30.3
	Other	42	6.5
	Retired	56	8.7
	Student	39	6.1
	Unemployed	94	14.6
Residence province	Eastern province	180	28.0
	Middle province	118	18.4
	Northern province	188	29.2
	Southern province	104	16.2
	Western province	53	8.2
Income	Less than 5,000	65	10.1
	5,000-9,999	160	24.9
	10,000-15,000	320	49.8
	More than 15,000	98	15.2
Location	Rural	83	12.9
	Suburban	321	49.9
	Urban	239	37.2
How many people you live with?	None	112	17.4
	1-3	307	47.7
	4-6 people	168	26.1
	More than 6 people	56	8.7

Table 2 shows diabetes-related information and glucagon knowledge data. A majority had T2DM (n=454, 70.6%), while 29.4% had T1DM (n=189). In terms of diabetes duration, over half had lived with the condition for one to five years (n=328, 51.0%), 28.1% for less than one year (n=181), 12.6% for six to 10 years (n=81), and 8.2% for more than 10 years (n=53). Insulin usage was evenly split, with 49.9% using insulin (n=321) and 50.1% not using it (n=322). Among insulin users, 19.0% had initiated therapy within the past year (n=122), 25.3% between one and five years ago (n=163), 4.4% between six and 10 years ago (n=28), and 4.2% over 10 years ago (n=27). Daily injection frequency varied as follows: 22.9% administered insulin twice daily (n=147), 16.6% once daily (n=107), 7.6 % three times (n=49), 4.2% four times (n=27), and 1.6% more than four times (n=10). Regarding hypoglycemia, 58.3% had never experienced low blood glucose (n=375) and 43.2% had never experienced severe episodes (n=278). Annual episode frequency was none for 28.3% (n=182), once for 36.2% (n=233), two to four times for 27.8% (n=179), five to nine times for 5.4% (n=35), and more than 10 times for 2.2% (n=14). Only 30.8% of all respondents (n=198) knew what a glucagon injection is; among them, 65.7% had glucagon at home (n=130), 82.3% knew how to use it (n=163), and 22.9% had it recommended by their doctor (n=147).

**Table 2:** Diabetes-related information and glucagon knowledge data among participants. DM: diabetes mellitus

Variable	Category	n	%
Diabetes type	T1D	189	29.4
	T2D	454	70.6
Time since diagnosis of DM	Less than 1 year	181	28.1
	1-5 years	328	51.0
	6-10 years	81	12.6
	More than 10 years	53	8.2
Using insulin	No	322	50.1
	Yes	321	49.9
Time to insulin use	Less than 1 year	122	19.0
	1-5 years	163	25.3
	6-10 years	28	4.4
	More than 10 years	27	4.2
Insulin per day	Once a day	107	16.6
	Twice a day	147	22.9
	3 times a day	49	7.6
	4 times	27	4.2
	More than 4 times	10	1.6
Have you experienced low blood glucose?	No	268	41.7
	Yes	375	58.3
Have you experienced severely low blood glucose?	No	365	56.8
	Yes	278	43.2
Low blood glucose episodes per year	None	182	28.3
	Once	233	36.2
	2-4 episodes	179	27.8
	5-9 episodes	35	5.4
	More than 10 episodes	14	2.2
Do you know what glucagon injection is?	No	445	69.2
	Yes	198	30.8
Do you have glucagon injection at home? (n=198)	No	68	34.3
	Yes	130	65.7
Do you know how to use glucagon? (n=198)	No	35	17.7
	Yes	163	82.3
Did your doctor recommend glucagon? (n=198)	No	51	25.8
	Yes	147	22.9

Table 3 shows the relationship between various demographic and health-related factors and respondents’ knowledge. Age played a significant role, with younger individuals (under 18 and 18-24 years) showing higher levels of glucagon knowledge (p<0.05). Gender and marital status did not appear to significantly affect glucagon knowledge. Education level had a notable effect, especially for those with a bachelor’s degree, who showed greater knowledge of glucagon (p<0.05). Employment status was strongly correlated with glucagon knowledge, with employed individuals being more likely to be familiar with and use glucagon (p<0.001). Income also played a role, as individuals earning 10,000 to 15,000 were more likely to know about glucagon compared to other income groups (p<0.05). Diabetes type significantly influenced glucagon knowledge, with T1DM patients being more likely to know about glucagon compared to T2DM patients (p<0.05). Finally, the duration of diabetes was also a significant factor, as those with diabetes for less than one year or one to five years were less likely to be knowledgeable compared to those with a diabetes duration of six to 10 years or more than 10 years (p<0.05).

**Table 3:** Association between glucagon knowledge-related variables and study variables.

Variable	Category	Do you know what a glucagon injection is?		
		Yes (n)	No (n)	p-Value
Age	Under 18 years	40	58	0.025
	18-24 years	69	155	
	24-34 years	30	116	
	35-44 years	23	45	
	45-54 years	23	38	
	55-64 years	8	25	
	65 years and above	5	8	
Gender	Male	121	267	0.79
	Female	77	178	
Marital status	Single	97	195	0.2
	Married	70	188	
	Divorced	7	22	
	Widowed	24	40	
Education level	Bachelor’s degree	94	240	0.11
	Diploma	29	74	
	Doctorate or higher	15	19	
	High school or less	29	66	
	Master’s degree	31	46	
Employment	Employed full time	61	156	<0.001
	Employed part time	58	137	
	Other	9	33	
	Retired	19	37	
	Student	26	13	
	Unemployed	25	69	
Income	10,000-15,000	86	234	0.039
	5,000-9,999	51	109	
	Less than 5,000	29	36	
	More than 15,000	32	66	
Location	Rural	28	55	<0.001
	Suburban	77	244	
	Urban	93	146	
Diabetes type	T1DM	69	120	0.04
	T2DM	129	325	
Duration of diabetes	Less than 1 year	50	131	0.02
	1-5 years	92	236	
	6-10 years	33	48	
	More than 10 years	23	30	
Using insulin	No	45	277	<0.001
	Yes	153	168	

## Discussion

The findings of the present study showed that diabetic patients in Riyadh, Saudi Arabia, had a lower knowledge level about glucagon. The results of the present study are significant, as there is a paucity of research on knowledge about glucagon among diabetic patients in Saudi Arabia. The findings of the present study are in line with the global trend of glucagon underutilization and poor education about emergency treatment, as only 30.8% of participants were aware of glucagon. These results are similar to AlGamdi et al., who reported that patients do not receive proper medication use education, especially lacking in the education of non-insulin therapies, showing a broader lack of diabetes education, including emergency treatments such as glucagon [17].

The results found that the T1DM patients in our study were notably more informed and more prone to having glucagon than their T2DM counterparts. This is in line with findings by Addala et al., who highlighted the disparities in diabetes technology access as well as education (specifically for the younger population and T1DM population) [18]. This is also in agreement with LaManna et al., who found that structured diabetes education improves hypoglycemia outcomes but provides a benefit that is more feasible for T1DM patients [19]. The age of the subjects also played a role, younger subjects had more awareness about glucagon. This is congruent with the study by Muradoğlu et al., who noted that younger patients and caregivers were engaged in hypoglycemia preparedness but still had anxiety related to glucagon use [20]. However, Seaquist et al. suggested that in real-world T1DM settings, the youth could have better exposure to the new administration, which may influence the correlation of youth with higher familiarity with glucagon [21]. Another reason for a higher knowledge level in the younger population is greater access to educational resources and their familiarity with digital tools. In contrast, older populations displayed lower glucagon knowledge, which is a concerning trend, especially since older diabetic patients are at a higher risk for hypoglycemic events and are often less likely to be actively engaged with current diabetes care methods.

Furthermore, glucagon awareness was also positively associated with education and employment status. Participants who had higher education levels or worked were significantly more likely to have knowledge of glucagon. The findings are consistent with Karagiannis et al., who investigated socioeconomic barriers to access advanced diabetes treatments and the importance of education and economic empowerment [22]. Similarly, Eberly et al. found huge imbalances in glucagon-like therapies depending on the level of education and wealth, as awareness was more common in well-educated and financially affluent individuals [23].

Haile and Fenta have reported that diabetic emergencies come with significant economic burdens in developing countries [24]. Schillinger reinforced the dire necessity of dealing with disparities based on socioeconomic factors, given the systemic complacency that allowed the inequities in life-saving diabetes care [25]. One of the interesting facts the study found was that there was no significant difference in physician recommendations of glucagon by income group. This contrasts with observations of Stuckey et al., who show that most of the patients and caregivers were unaware of glucagon, often because the physicians did not discuss it [26]. Similarly, Alqifari et al. also found differences in diabetes care and complication rates between urban and rural settings in Saudi Arabia, which is also seen in the present study, that urban residents had higher glucagon awareness and access as compared to rural residents [27].

Access to insulin and the duration of diabetes diagnosis were also linked to glucagon awareness. Our findings showed that insulin users were more likely to have been prescribed or educated about glucagon, consistent with Kallem et al., who underscored that insulin users are at heightened hypoglycemia risk and thus more in need of glucagon [28]. However, there were still knowledge gaps among insulin users, as Stuckey et al. reported that insulin-treated patients failed to recognize the need for glucagon when they misperceived or did not discuss it with healthcare providers [26]. Additionally, patients with diabetes diagnosed in the past five years were more aware of glucagon than those with diabetes for more than five years. This is consistent with the study by Allweiss that newly diagnosed patients, especially those affected by natural disasters or healthcare disruptions, need to be considered emergency cases [29]. LaManna et al. also suggested that there should be a greater focus on early-stage education, with follow-up less probable, resulting in knowledge attrition [19].

Lastly, the digital divide also appears to play a role in access to and education on glucagon. Ebekozien et al. point out that diabetes technology, such as CGMs and emergency glucagon, has inequities in access, further worsening existing healthcare disparities [30]. Niskanen et al. also highlighted that disparities in glycemic control and treatment intensification tend to creep further over time in under-resourced populations [31].

Collectively, these observations reflect the local and international studies according to which the socioeconomic disparities, educational inadequacies, and insufficient physician-patient communication have all resulted in the remarkably neglected clinical use of glucagon. Future interventions should aim to increase public awareness, provide essential tools to healthcare providers to educate patients, and ensure equal access between groups of different demographics and economics.

Limitations

There are several limitations of the study that should be considered while interpreting the findings of the study. The main limitation is the inability to fully identify and analyze the determinants influencing participants' knowledge and use of glucagon, as the study design and data collection tools may not have captured all relevant factors. Furthermore, data were collected via a self‑administered questionnaire, which introduces potential sources of bias, including recall bias. Third, the sampling strategy relied on convenience recruitment through social media, support groups, and healthcare facilities, and although the target sample size calculation accounted for non‑response, the final sample may not be representative of the broader diabetic population in Riyadh or Saudi Arabia. Another limitation of this study is digital access bias, which could have led to underrepresentation of certain demographic groups, especially the older population.

## Conclusions

The findings of the present study showed that there is a lack of knowledge about glucagon use among diabetic patients in Riyadh, Saudi Arabia. The findings reveal that a significant majority of participants are either unaware of glucagon’s existence or do not have access to it, which poses a critical challenge in managing severe hypoglycemic episodes. However, younger individuals, those with higher education, and employed persons demonstrated a higher level of glucagon knowledge. This illustrates that sociodemographic factors can play a role in knowledge about glucagon. These findings also necessitate that all patients with diabetes should be given awareness about glucagon and its use in emergency conditions. Furthermore, persons related to diabetic patients should also be given knowledge about glucagon use to help manage episodes of hypoglycemia.

## Appendices

**Table 4:** Questionnaire used in this study.

No.	Question	Response options
1	Age in years	Numeric value (years)
2	Gender	◻ Male ◻ Female
3	Marital status	◻ Single ◻ Married ◻ Divorced ◻ Widowed
4	Living area	◻ Rural ◻ Suburban ◻ Urban
5	Duration of diabetes	◻ 10 years
6	Duration of insulin use	◻ Not using insulin ◻ 10 years
7	Insulin injections per day	◻ Once ◻ Twice ◻ 3 times ◻ 4 times ◻ >4 times
8	Last HbA1c	Numeric value (%)
9	Body height	Numeric value (cm)
10	Body weight	Numeric value (kg)
11	Education level	◻ High school or less ◻ Diploma ◻ Bachelor’s degree ◻ Master’s degree ◻ Doctorate or higher
12	Number of household members	◻ Living alone ◻ 1-3 people ◻ 4-6 people ◻ >6 people
13	Monthly income	◻ 15,000
14	Type of diabetes	◻ Type 1 diabetes ◻ Type 2 diabetes
15	Ever had an episode of hypoglycemia? (weakness, tremors, light‑headedness)	◻ Yes ◻ No
16	Ever had an episode of severe hypoglycemia? (reported by others: unconsciousness, seizure)	◻ Yes ◻ No
17	Do you know what a glucagon injection is?	◻ Yes ◻ No
18	Do you have glucagon injection available at home?	◻ Yes ◻ No
19	Do you know how to use glucagon injection?	◻ Yes ◻ No
20	Was glucagon recommended to you by your family physician?	◻ Yes ◻ No
21	Frequency of hypoglycemia episodes per year	◻ None ◻ Once ◻ 2-4 episodes ◻ 5-9 episodes ◻ >10 episodes
